# Prospective Survey of Financial Toxicity Measured by the Comprehensive Score for Financial Toxicity in Japanese Patients With Cancer

**DOI:** 10.1200/JGO.19.00003

**Published:** 2019-05-09

**Authors:** Kazunori Honda, Bishal Gyawali, Masashi Ando, Ryosuke Kumanishi, Kyoko Kato, Keiji Sugiyama, Seiichiro Mitani, Toshiki Masuishi, Yukiya Narita, Hideaki Bando, Hiroya Taniguchi, Shigenori Kadowaki, Takashi Ura, Kei Muro

**Affiliations:** ^1^ Aichi Cancer Center Hospital, Nagoya, Japan; ^2^ Queen’s University, Kingston, Ontario, Canada

## Abstract

**PURPOSE:**

We previously reported on the pilot study assessing the feasibility of using the Japanese translation of the Comprehensive Score for Financial Toxicity (COST) tool to measure financial toxicity (FT) among Japanese patients with cancer. In this study, we report the results of the prospective survey assessing FT in Japanese patients with cancer using the same tool.

**PATIENTS AND METHODS:**

Eligible patients were receiving chemotherapy for a solid tumor for at least 2 months. In addition to the COST survey, socioeconomic characteristics were collected by using a questionnaire and medical records.

**RESULTS:**

Of the 191 patients approached, 156 (82%) responded to the questionnaire. Primary tumor sites were colorectal (n = 77; 49%), gastric (n = 39; 25%), esophageal (n = 16; 10%), thyroid (n = 9; 6%), head and neck (n = 4; 3%), and other (n = 11; 7%). Median COST score was 21 (range, 0 to 41; mean ± standard deviation, 12.1 ± 8.45), with lower COST scores indicating more severe FT. On multivariable analyses using linear regression, older age (β, 0.15 per year; 95% CI, 0.02 to 0.28; *P* = .02) and higher household savings (β, 8.24 per ¥15 million; 95% CI, 4.06 to 12.42; *P* < .001) were positively associated with COST score; nonregular employment (β, −5.37; 95% CI, −10.16 to −0.57; *P* = .03), retirement because of cancer (β, −5.42; 95% CI, −8.62 to −1.37; *P* = .009), and use of strategies to cope with the cost of cancer care (β, −5.09; 95% CI, −7.87 to −2.30; *P* < .001) were negatively associated with COST score.

**CONCLUSION:**

Using the Japanese version of the COST tool, we identified various factors associated with FT in Japanese patients with cancer. These findings will have important implications for cancer policy planning in Japan.

## INTRODUCTION

Financial toxicity (FT) in cancer care refers to the downstream detrimental effects on the financial well-being of patients and families as a result of a cancer diagnosis.^1^ FT, also referred to as economic burden, economic hardship, financial burden, financial distress, financial hardship, and financial stress, thus represents a broad concept including multifaceted influences of cancer treatment on the financial well-being of patients. Previously thought of as a problem of low- and middle-income countries, where many patients cannot afford treatment,^2^ FT has now become a serious issue even in high-income countries. There are several reports from high-income countries suggesting a negative association between FT and both length and quality of life.^3-5^

Japan has a public medical insurance system.^6^ Under the Japanese universal health care system, every citizen or foreign resident is compulsorily required to join the national health insurance, which pays for 70% of all health care costs. The remaining 30% of health care costs are paid by the patients out of pocket. The Japanese health care system also has some additional protections against FT. For example, a patient older than age 75 years is required to pay only 10% of his or her health care bills out of pocket instead of the usual 30%. In addition, there is a unique system of a ceiling amount for high-cost medical expenses. This limit differs with the patient’s age and income, ranging from ¥10,000 to ¥250,000 per month (approximately US$90 to US$2,270). This ceiling amount is the maximum amount any patient has to pay from his or her pocket. That means if a patient’s out-of-pocket medical care bill exceeds the ceiling amount, all costs beyond the ceiling amount are covered with public subsidies. Logically, the public health system that pays for treatment costs of patients should protect patients from financial burden. However, despite such protections, in a survey of Japanese patients using imatinib for chronic myeloid leukemia, 75.8% of patients felt financial burden and 31.7% considered discontinuation of therapy because of the financial burden. Some patients (2.6%) even temporarily stopped their imatinib prescription because of financial burden.^7^ Such FT among patients with cancer has also been reported from countries like the United Kingdom and Italy,^4,8^ where the government pays for 100% of health care costs.

The Comprehensive Score for Financial Toxicity (COST) score has been validated as a useful tool for measuring FT among patients with cancer in the United States.^9,10^ We have previously reported on the pilot study in which we assessed the feasibility of using the Japanese version of the COST questionnaire to measure FT among Japanese patients with cancer.^11^ Here, we report on the burden and characteristics of FT among Japanese patients with cancer using the same tool.

## PATIENTS AND METHODS

### Patients

Patients receiving ongoing chemotherapy in Aichi Cancer Center Hospital, a public regional cancer center in Nagoya, Japan, were recruited. Eligibility criteria included patients receiving anticancer drug therapy (chemotherapy, targeted therapy, or immunotherapy) for at least 2 months, age 20 years or older, with the ability to read and write in Japanese. Patients receiving neoadjuvant or adjuvant chemotherapy were also eligible to participate if the eligibility criteria were met.

### Procedure

Eligible patients provided informed consent and were handed the Japanese version of the COST questionnaire, which they could fill out at home and send to us by mail. Prepaid postal envelopes were provided to the patients using the institutional funds. No other funding support was received for this study. The study was approved by the institutional review board of Aichi Cancer Center Hospital. Patients did not receive any financial assistance for participation in this study.

### Data Collection and Analyses

Questionnaire items included the Japanese translation of the COST score, out-of-pocket medical costs, total family income (six categories at ¥2 million intervals), total family savings (seven categories at ¥2 million intervals; US$1 = approximately ¥110). Information on socioeconomic background was also collected and included marital status, household size, educational status, work status, and total assets. Information on disease status was obtained from the electronic medical record and included primary tumor site, local versus metastatic tumors, duration of chemotherapy, and chemotherapy regimen at the time of questionnaire.

The COST score is an 11-item patent-reported outcomes measure used to evaluate FT. Every item ranges in score from 0 to 4; thus, the total COST score can range from 0 to 44, with a lower score representing a greater degree of FT. Accordingly, a score of 0 represents the highest FT, and a score of 44 represents the lowest FT.

### Statistical Analyses

Patient characteristics are summarized using descriptive statistics. Continuous variables are expressed as medians (ranges), and categorical variables are expressed as frequencies (percentages). After selection of covariates in univariable analyses (*P* < .10) and previous reports, multivariable analyses were performed using a linear regression model to assess the independent factors associated with COST score. The correlation coefficient (β) and 95% CI were calculated. Sample size of 150 was considered adequate, because we expected 10 to 15 covariates in multivariable analyses. We decided to approach patients until we received at least 150 responses. All calculations were performed using R statistical software (version 3.3.2; R Foundation for Statistical Computing, Vienna, Austria), and two-sided *P* values less than .05 were considered statistically significant.

## RESULTS

Of the 191 patients approached, 156 (82%) responded to the questionnaire. Median age of the respondents was 67 years (range, 30 to 87 years), and 83 patients (53%) were men (Table 1). Patients were receiving treatment for colorectal (n = 77; 49%), gastric (n = 39; 25%), esophageal (n = 16; 10%), thyroid (n = 9; 6%), head and neck (n = 4; 3%), or other cancers (including breast, melanoma, or sarcoma; n = 11; 7%). Ninety-six patients (62%) were receiving treatment with at least one molecular targeted agent, including bevacizumab, trastuzumab, cetuximab, panitumumab, ramucirumab, sunitinib, sorafenib, pazopanib, imatinib, lenvatinib, regorafenib, or everolimus. No patients were receiving immunotherapy. Median duration from the start of chemotherapy was 12 months (range, 2 to 138 months). The most frequent category of annual household income was between ¥2 million to ¥4 million, and household savings was more than ¥15 million. Twenty-nine patients (19%) had to retire from their work because of cancer.

**TABLE 1:**
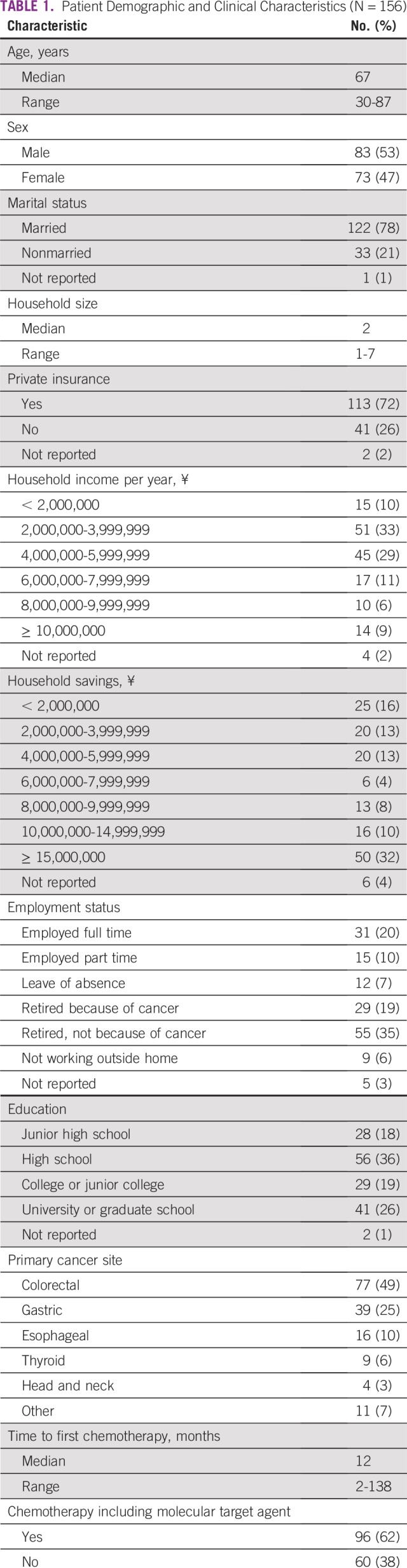
Patient Demographic and Clinical Characteristics (N = 156)

**FIG 1:**
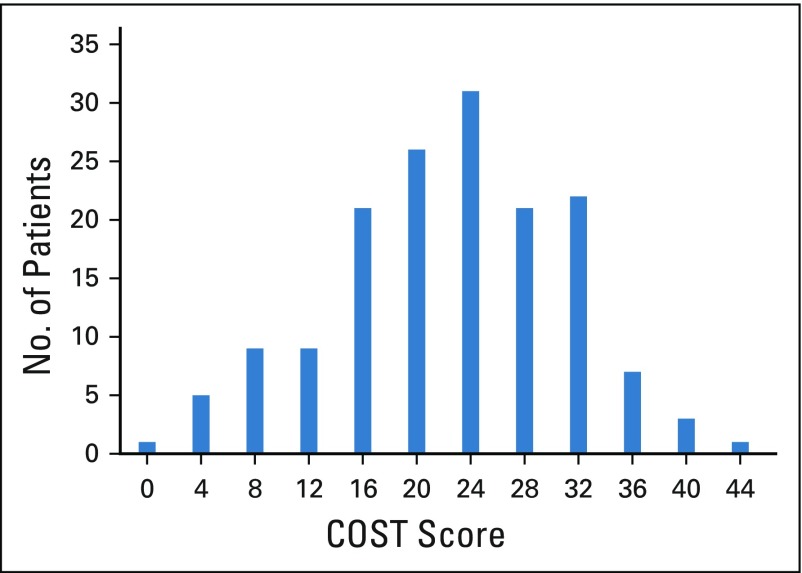
Distribution of Comprehensive Score for Financial Toxicity (COST) score showed normal distribution curve.

The distribution of COST score is listed in Table 2 and Figure 1. Median COST score was 21 (range, 0 to 41; mean ± standard deviation, 12.1 ± 8.45). Strategies to cope with the cost of cancer care included using savings to pay for cancer treatment (n = 95; 63%), cutting spending on leisure (n = 67; 44%), and cutting spending on food or clothing (n = 42; 28%; Table 3). On multivariable analyses associated with COST score using linear regression, older age (β, 0.15 per year; 95% CI, 0.02 to 0.28; *P* = .02) and higher household savings (β, 8.24 per ¥15 million; 95% CI, 4.06 to 12.42; *P* < .001) were significantly associated with higher COST score, which indicates lower FT (Table 4). Nonregular employment (β, −5.37; 95% CI, −10.16 to −0.57; *P* = .03), retirement because of cancer (β, −5.42; 95% CI, −8.62 – −1.37; *P* = .009), and use of strategies to cope with the cost of cancer care expenses (β, −5.09; 95% CI, −7.87 to −2.30; *P* < .001) were significantly associated with lower COST score, which indicates higher FT.

**TABLE 2:**
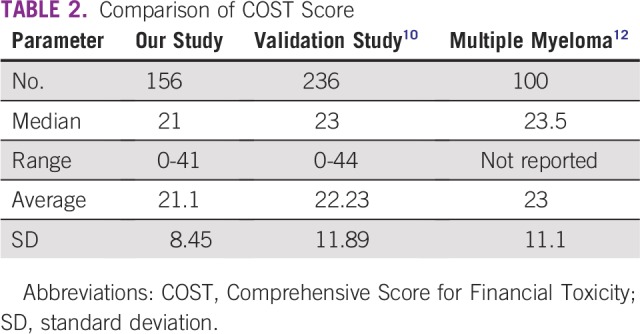
Comparison of COST Score

**TABLE 3:**
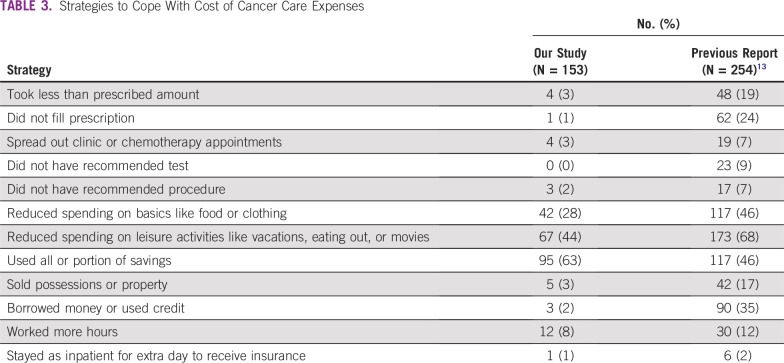
Strategies to Cope With Cost of Cancer Care Expenses

**TABLE 4:**
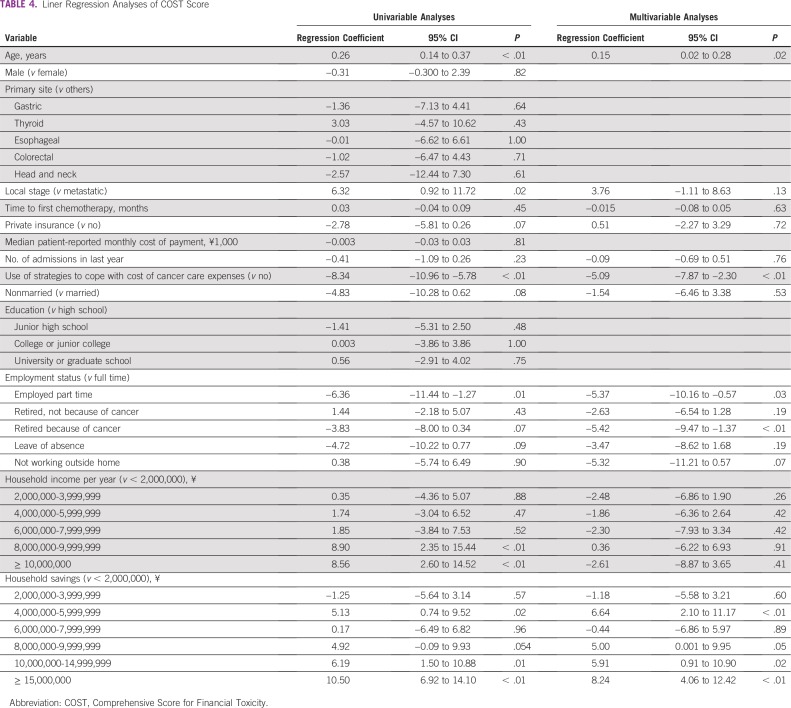
Liner Regression Analyses of COST Score

## DISCUSSION

To our knowledge, this is the first report to evaluate the factors associated with FT in patients with cancer in Japan. Using the COST questionnaire, we showed that more than 60% of patients used some alternative strategies, such as cutting spending on food, clothing, or leisure to cope with FT. These results show that a substantial proportion of Japanese patients with cancer have experienced FT despite the universal insurance system in place.

The COST tool was developed by de Souza et al^9,10^ to objectively quantify FT in patients with cancer. This tool has already been validated in patients with advanced cancer in the United States.^9^ Huntington et al^12^ applied this COST tool to measure FT in 100 patients with multiple myeloma and found that the COST tool successfully evaluated FT and was correlated with the use of strategies to cope with treatment expenses, such as borrowing money. However, it was unknown whether the same tool could be used in other countries with different health care or medical insurance systems. In previous reports of the COST questionnaire from the United States, median COST scores were 23.5 and 23,^10,12^ which are similar to the score of 21 in our study.

Several studies using the COST tool from the United States have reported the association of COST score with race, employment status, number of hospital admissions, age, marital status, time from diagnosis, income, and medical expenses.^10,12^ In a systematic review of studies where 85% of patients with cancer were from the United States, FT was observed in 28% to 48% of patients using objective measures and 16% to 73% of patients using subjective measures. Female sex, younger age, low income at baseline, adjuvant therapy, and more recent diagnosis were associated with greater susceptibility to FT.^14^ Our study showed that COST score in Japanese patients with cancer was significantly associated with younger age, lower household savings, nonregular employment, retirement because of cancer, and use of strategies to cope with the cost of cancer care expenses.

There are some interesting differences between factors affecting FT in Japan and the United States on the basis of differences in medical systems and cultures. In the United States, the time from diagnosis and the number of hospital admissions influenced COST score; however, any factor directly related to treatment was not significantly associated with FT in our study. In Japan, COST score was more associated with income-related factors than treatment-related factors, probably because of the public health insurance system in Japan, which is different to the situation in the United States. Nevertheless, many patients with cancer in Japan seem to experience FT. This finding is in line with the reports of FT from countries with existing public insurance systems, such as the United Kingdom and Italy,^4,8^ suggesting that the self-pay amount of medical expenses is not the only factor leading to FT.

Financial burden in patients with cancer can result from either increased expenditures or reduced assests.^15^ Expenditures includes drug costs, other medical expenses, and costs indirectly related to treatment (eg, transportation expenses). The assets consist of income sources, such as salary and savings. In Japan, the proportion of assets seems to have a larger effect on FT than the proportion of expenditures, unlike the scenario in the United States.

FT is severe in younger patients, both in Japan and the United States. In general, younger people have lower incomes, with relatively small savings, whereas their expenses are higher, because many of them have small children or educational loans. However, the Japanese insurance system gives preferential treatment to elderly patients. Our findings of more severe FT in younger patients with cancer and income-related factors affecting FT more than treatment-related factors will have important policy implications for policymakers in Japan.

FT has been shown to be associated with bankruptcy,^5^ discontinuation of therapy, poor drug adherence, and refusal of necessary care,^16-18^ ultimately affecting survival^4^ and quality of life^15^ in multiple studies. Therefore, objective measurement and quantification of FT are essential for proper policy planning. Studies from Japan assessing the prevalence and consequences of FT in patients with cancer are lacking. We believe this study can contribute to greater understanding of FT among Japanese patients with cancer and provide useful data for cancer policy planning.

There are several limitations in this research. This study was performed at a single department in a single institution. Patients who participated in our survey lived in and around a relatively urban city, Nagoya, which is also reflected in the relatively larger assets our patients had compared with the Japanese median. A majority of our patients had GI cancer. There is also a risk of responder bias in our study, because the patients who responded to this survey by default were under continued treatment, meaning that the FT was not severe enough to cause discontinuation of therapy. No patients in our study received immunotherapy. Our study, therefore, may have underestimated the prevalence of FT among patients with cancer in Japan. There is also a possibility of underclaiming or overclaiming of income or property. Furthermore, 18% of patients in our survey were older than age 75 years; such patients pay only 10% of their health care bills out of pocket and would experience a lesser degree of FT. However, mean COST scores for these patients were not different from those younger than age 75 years (23.1 *v* 20.6; *P* = .167). We need to conduct a similar survey across the country and include all tumor sites proportionately to produce generalizable results. Finally, we did not investigate whether FT was different between patients receiving high-value versus low-value care.^19^ Future studies exploring the relation between receipt of value-based care and FT are needed.

Using the Japanese version of the COST tool, we identified various factors associated with FT in Japanese patients with cancer. These findings will have important implications for cancer policy planning in Japan.
